# Transcriptome profiles of high-lysine adaptation reveal insights into osmotic stress response in *Corynebacterium glutamicum*

**DOI:** 10.3389/fbioe.2022.933325

**Published:** 2022-08-09

**Authors:** Jian Wang, Jian Yang, Guoxin Shi, Weidong Li, Yun Ju, Liang Wei, Jun Liu, Ning Xu

**Affiliations:** ^1^ College of Biological and Agricultural Engineering, Jilin University, Changchun, China; ^2^ School of Food Engineering and Biotechnology, Tianjin University of Science and Technology, Tianjin, China; ^3^ Tianjin Institute of Industrial Biotechnology, Chinese Academy of Sciences, Tianjin, China; ^4^ Key Laboratory of Systems Microbial Biotechnology, Chinese Academy of Sciences, Tianjin, China

**Keywords:** transcriptome profiles, high-lysine stress, molecular chaperon, DNA repair, osmoadaptation, *C. glutamicum*

## Abstract

*Corynebacterium glutamicum* has been widely and effectively used for fermentative production of l-lysine on an industrial scale. However, high-level accumulation of end products inevitably leads to osmotic stress and hinders further increase of l-lysine production. At present, the underlying mechanism by which *C. glutamicum* cells adapt to high-lysine-induced osmotic stress is still unclear. In this study, we conducted a comparative transcriptomic analysis by RNA-seq to determine gene expression profiles under different high-lysine stress conditions. The results indicated that the increased expression of some metabolic pathways such as sulfur metabolism and specific amino acid biosynthesis might offer favorable benefits for high-lysine adaptation. Functional assays of 18 representative differentially expressed genes showed that the enhanced expression of multiple candidate genes, especially *grpE* chaperon, conferred high-lysine stress tolerance in *C. glutamicum*. Moreover, DNA repair component MutT and energy-transducing NADH dehydrogenase Ndh were also found to be important for protecting cells against high-lysine-induced osmotic stress. Taken together, these aforementioned findings provide broader views of transcriptome profiles and promising candidate targets of *C. glutamicum* for the adaptation of high-lysine stress during fermentation.

## Introduction

l-Lysine is an essential amino acid that has been widely used in the animal feed, cosmetic, and pharmaceutical industries (Cheng et al., 2018). Currently, the worldwide production of lysine salts is estimated to be 2.5 million tons with a growth rate of 7% per year (Ikeda, 2017; Pandey et al., 2020). In recent years, the production of l-lysine has been almost exclusively by microbial fermentation, which has been accomplished with diverse engineered strains including *Corynebacterium glutamicum, Escherichia coli*, and *Brevibacterium flavum* (Ikeda, 2017)*.* However, during industrial fermentation processes, the microorganisms are often exposed to multiple harsh biotic and abiotic factors such as pH, temperature, and osmolarity. Of them, metabolite-induced osmotic stress often causes serious damages to cellular fitness and lysine accumulation (Rönsch et al., 2003; Bremer and Krämer, 2019).

As an important GRAS (generally regarded as safe) microorganism, *C. glutamicum* has been well-established for the industrial production of lysine for more than 50 years (Ikeda and Takeno, 2013; Wendisch et al., 2016; Ikeda, 2017). Previous attempts at strain improvement for l-lysine production have mainly relied on the regulation of specific metabolic pathways via traditional mutagenesis or metabolic engineering. In some exciting cases, *C. glutamicum* has been successfully engineered to produce high levels of l-lysine above 200 g/L in fed-batch fermentation (Xu et al., 2019; Xu et al., 2020). However, the accumulation of large amounts of l-lysine during fermentation inevitably triggers hyperosmotic stress on the microbial workhorse, thus hindering further improvement of l-lysine production (Wang et al., 2019). To overcome such limitations, an improved understanding of cellular metabolism and the physiology of *C. glutamicum* in response to high-lysine-induced osmotic stress may provide a new basis for generating robust cell factories with desired traits.

In past decades, engineering the robustness of strains to industrially relevant stress factors has proven to be conducive to achieving higher yield and productivity (Mukhopadhyay, 2015; Ko et al., 2020; Liu et al., 2021; Mohedano et al., 2022). However, the underlying mechanisms of how *C. glutamicum* tolerates high-lysine concentrations are still unclear, which obviously impedes the identification of promising targets for improving the robustness of cell factories. To gain new insights into the genome-wide transcriptional profiling of *C. glutamicum* under short- and long-term high-lysine stress conditions, we performed comparative RNA-seq transcriptomic analyses aimed at investigating potential genes associated with lysine-induced osmotic stress responses. The results revealed that the presence of high levels of l-lysine greatly increased the expression of several metabolic pathways such as sulfur metabolism, arginine and proline biosynthesis, and folate biosynthesis, whereas the central carbon metabolic pathways, especially glycolysis, were impaired to varying degrees. Functional analysis of 18 differentially expressed genes showed that the enhanced expression of multiple candidate genes, especially *grpE* chaperon, conferred high-lysine tolerance. In addition, we also found that the presence of DNA repair component MutT and respiratory chain NADH dehydrogenase Ndh were essential for osmoadaptation in *C. glutamicum*, which enabled cells to protect themselves against high-lysine-induced osmotic stress. Overall, these aforementioned findings contributed to giving a global view of the transcriptome profiles of *C. glutamicum* in the adaptation of high-lysine stress during fermentation.

## Materials and methods

### Strains and growth conditions

Bacterial strains and plasmids used in this study are listed in Table 1. *E. coli* DH5*α* was used as host cells for general cloning. *C. glutamicum* ATCC 13032 was used as the parental strain for genetic manipulation and the wild-type strain for functional analysis. Unless otherwise specified, *E. coli* cells were routinely grown at 37°C in LB medium (0.5% yeast extract, 1% tryptone, and 1% NaCl), and *C. glutamicum* cells were cultivated at 32°C in CGXII minimal medium (50 g/L glucose, 20 g/L (NH_4_)_2_SO_4_, 5 g/L urea, 1 g/L KH_2_PO_4_, 1 g/L K_2_HPO_4_, 0.25 g/L MgSO_4_•7H_2_O, 42 g/L 3-morpholinopropanesulfonic acid, 10 mg/L CaCl_2_, 10 mg/L FeSO_4_•7H_2_O, 10 mg/L MnSO_4_•H_2_O, 1 mg/L ZnSO_4_•7H_2_O, 0.2 mg/L CuSO_4_, 0.02 mg/L NiCl_2_•6H_2_O, 0.2 mg/L biotin, and 0.03 g/L protocatechuic acid). When required, antibiotics were added to the media at a final concentration of 15 μg/L chloramphenicol or 25 μg/L kanamycin for *C. glutamicum*, and 20 μM isopropyl β-d-1-thiogalactopyranoside (IPTG) was used for the induced expression of target genes.

**TABLE 1:** Strains and plasmids used in this study.

Plasmid or strain	Description	Source
Plasmids		
pCRD206	Temperature-sensitive replicon and *B. subtilis sacB* gene, Kan^r^	Okibe et al. (2011)
pXMJ19	*C. glutamicum*-*E. coli* shuttle expression vector, Cm^r^	Lab stock
pECXK-99E	*C. glutamicum*-*E. coli* shuttle expression vector, Kan^r^	Lab stock
pXMJ19-*cg0074*	pXMJ19 derivative, containing the *cg0074* gene from *C. glutamicum*	This study
pXMJ19-*cg1152*	pXMJ19 derivative, containing the *cg1152* gene from *C. glutamicum*	This study
pXMJ19-*cg0767*	pXMJ19 derivative, containing the *cg0767* gene from *C. glutamicum*	This study
pXMJ19-*cg2313*	pXMJ19 derivative, containing the *cg2313* gene from *C. glutamicum*	This study
pXMJ19-*cg1424*	pXMJ19 derivative, containing the *cg1424* gene from *C. glutamicum*	This study
pXMJ19-*cg0470*	pXMJ19 derivative, containing the *cg0470* gene from *C. glutamicum*	This study
pXMJ19-*cg0785*	pXMJ19 derivative, containing the *cg0785* gene from *C. glutamicum*	This study
pXMJ19-*cg0362*	pXMJ19 derivative, containing the *cg0362* gene from *C. glutamicum*	This study
pXMJ19-*cg2919*	pXMJ19 derivative, containing the *cg2919* gene from *C. glutamicum*	This study
pXMJ19-*cg1379*	pXMJ19 derivative, containing the *cg1379* gene from *C. glutamicum*	This study
pXMJ19-*dnaJ*	pXMJ19 derivative, containing the *dnaJ* gene from *C. glutamicum*	This study
pXMJ19-*grpE*	pXMJ19 derivative, containing the *grpE* gene from *C. glutamicum*	This study
pXMJ19-*dnaK*	pXMJ19 derivative, containing the *dnaK* gene from *C. glutamicum*	This study
pXMJ19-*groES*	pXMJ19 derivative, containing the *groES* gene from *C. glutamicum*	This study
pXMJ19-*groEL*	pXMJ19 derivative, containing the *groEL* gene from *C. glutamicum*	This study
pXMJ19-*dps*	pXMJ19 derivative, containing the *dps* gene from *C. glutamicum*	This study
pXMJ19-*cysIHDN*	pXMJ19 derivative, containing the *cysIHDN* gene from *C. glutamicum*	This study
pXMJ19-*ssuDCBA*	pXMJ19 derivative, containing the *ssuDCBA* gene from *C. glutamicum*	This study
pECXK-99E-*mutT*	pECXK-99E derivative, containing the *mutT* gene from *C. glutamicum*	This study
pECXK-99E-*ndh*	pECXK-99E derivative, containing the *ndh* gene from *C. glutamicum*	This study
Strains		
DH5α	*E. coli* derivative; competent cells for general cloning	Invitrogen
ATCC 13032	Representative wild-type *C. glutamicum* strain	Lab stock
*∆uvrA*	*C. glutamicum* mutant derivative; lacks the *uvrA* gene	This study
*∆xpb*	*C. glutamicum* mutant derivative; lacks the *xpb* gene	Lab stock
*∆mfd*	*C. glutamicum* mutant derivative; lacks the *mfd* gene	This study
*∆uvrD*	*C. glutamicum* mutant derivative; lacks the *uvrD* gene	This study
*∆ung*	*C. glutamicum* mutant derivative; lacks the *ung* gene	Lab stock
*∆mutM1*	*C. glutamicum* mutant derivative; lacks the *mutM1* gene	This study
*∆tagA2*	*C. glutamicum* mutant derivative; lacks the *tagA2* gene	This study
*∆mutY*	*C. glutamicum* mutant derivative; lacks the *mutY* gene	Lab stock
*∆n*th	*C. glutamicum* mutant derivative; lacks the *n*th gene	This study
*∆nei*	*C. glutamicum* mutant derivative; lacks the *nei* gene	This study
*∆mutT*	*C. glutamicum* mutant derivative; lacks the *mutT* gene	This study
*∆xseA*	*C. glutamicum* mutant derivative; lacks the *xseA* gene	Lab stock
*∆nucS*	*C. glutamicum* mutant derivative; lacks the *nucS* gene	Lab stock
*∆recJ*	*C. glutamicum* mutant derivative; lacks the *recJ* gene	This study
*∆recA*	*C. glutamicum* mutant derivative; lacks the *recA* gene	Lab stock
*∆recF*	*C. glutamicum* mutant derivative; lacks the *recF* gene	This study
*∆recG*	*C. glutamicum* mutant derivative; lacks the *recG* gene	This study
*∆cg1318*	*C. glutamicum* mutant derivative; lacks the *cg1318* gene	This study
*∆cg2228*	*C. glutamicum* mutant derivative; lacks the *cg2228* gene	This study
*∆recB*	*C. glutamicum* mutant derivative; lacks the *recB* gene	This study
*∆ndh*	*C. glutamicum* mutant derivative; lacks the *ndh* gene	This study
*∆mutT* + *mutT*	*C. glutamicum* mutant derivative; containing the pECXK-99E-*mutT* plasmid	This study
*∆ndh* + *ndh*	*C. glutamicum* mutant derivative; containing the pECXK-99E-*ndh* plasmid	This study

### Plasmid and strain construction

The *E. coli*-*C. glutamicum* shuttle vector pXMJ19 or pECXK-99E was typically used as the backbone for the construction of expression vector systems (Pátek and Nešvera, 2013). The coding regions of the target genes were amplified from the genomic DNA of *C. glutamicum* ATCC 13032 using the specific primers targeting corresponding genes. The primers used in this study are listed in Supplementary Table S1. The PCR fragments were then purified and subcloned into the backbone of the shuttle vector to generate the inducible expression recombinant plasmids according to the classic digestion-ligation or Golden Gate assembly methods. The expression vectors were then transformed into the wild-type strain *C. glutamicum* ATCC 13032 for subsequent functional analysis.

*C. glutamicum* gene deletion mutants were obtained by two-step homologous recombination based on the temperature-sensitive plasmid pCRD206 as previously described (Okibe et al., 2011). The primers used in this study are listed in Supplementary Table S1. To avoid the polar effects, the markerless chromosomal in-frame deletion mutants were constructed. For example, the *∆mutT* (*cg3043*) mutant was constructed as follows: the *mutT* upstream and downstream regions were amplified with Thermo Scientific Phusion High-Fidelity DNA Polymerase from *C. glutamicum* genomic DNA with the primer pairs mutT-1-For/mutT-2-Rev and mutT-3-For/mutT-4-Rev, respectively. These two PCR fragments were then assembled with the *Bam*HI/*Xba*I-linearized pCRD206 backbone to yield the corresponding deletion plasmid by a seamless cloning method using the ClonExpress^®^ II One Step Cloning Kit (Vazyme Biotech, China). The resulting plasmid was then introduced into *C. glutamicum* ATCC 13032 by electroporation, and it generated the chromosomal-disrupted mutant through a two-step selection strategy. For the complemented strains, a pECXK-99E-based plasmid carrying the targeting gene was transferred into the relevant mutants by electroporation. All *C. glutamicum* mutants were obtained by a similar strategy. The correct mutants were confirmed by colony-PCR and Sanger sequencing.

### Cell growth assay

The corresponding single colony was randomly picked from plates and incubated in a rich LBHIS medium (2.5 g/L yeast extract, 5 g/L tryptone, 5 g/L NaCl, 18.5 g/L brain heart infusion, and 91 g/L sorbitol) at 32°C for 16 h with shaking. Overnight cultures of indicated strains were then harvested and resuspended in a specific CGXII medium to an initial OD_600_ of 0.1 prior to growth experiments. Different concentrations of l-lysine were added into the medium to mimic the osmotic stress that occurs during the fermentation process. In order to determine growth abilities in response to different lysine levels, overnight cultures of the wild-type strain were transferred into a 100-ml flask containing 20 ml of fresh CGXII medium and grown at 32°C with shaking for 20 h. For determination of growth curves and preparation of RNA-seq samples, the growth experiments were carried out in 250 ml Erlenmeyer flasks containing 100 ml of CGXII medium at 30°C with shaking. Cell growth was monitored by measuring the optical density at 600 nm at the indicated time points and shown as averages of at least two independent repeats.

### RNA-seq transcriptome analysis

Overnight cultures of the *C. glutamicum* wild-type strain were inoculated into the fresh CGXII medium with and without 160 g/L l-lysine at an initial OD_600_ of 0.1, and cells were harvested for RNA extraction when midexponential growth was reached. For the non-lysine-treated WT samples, cells were collected by centrifugation after approximately 12 h of incubation, whereas the long-term lysine-treated HL samples were collected by centrifugation after constantly suffering from high-lysine stress for 18 h. In addition, the midexponential WT cultures were harvested and retreated with 160 g/L lysine stress for 1 h to obtain the short-term lysine-treated ST samples. For each group, two independent biological samples were estimated. Total RNA was isolated from *C. glutamicum* cells with the RNAprep Pure Cell/Bacteria Kit (Tiangen Biotech, Beijing, China). The assessment of RNA integrity was performed using the RNA Nano 6000 Assay Kit of the Agilent Bioanalyzer 2100 system (Agilent Technologies, Palo Alto, United States). cDNA library construction and Illumina sequencing were performed at the Beijing Novogene Bioinformatics Technology Co., Ltd. The raw data were filtered by removing reads containing adapters, reads containing poly-N, and low-quality reads and aligned to the reference genome of *C. glutamicum* ATCC 13032 using the Bowtie2 aligner (Langmead and Salzberg, 2012). RNA-seq raw data for this project were deposited in the NCBI SRA database under the accession number PRJNA835314. Gene expression was quantified using the RPKM algorithm (the reads per kilobase of transcript sequence per million mapped reads). RNA-seq differential expression analysis between two groups was performed using the DESeq2 R package (Love et al., 2014). *p*-values were adjusted for multiple testing using the Benjamini–Hochberg method with a false discovery rate of 5% to generate padj, and |log2(fold change)| > 0 & padj < 0.05 was set as the threshold for significant differential expression of genes (DEGs).

### Lysine-induced osmotic tolerance assay

For the growth tolerance experiments, 160 g/L l-lysine was used to mimic osmotic stress that often occurs during the middle or late stage of industrial fermentation. Single colonies of wild-type and mutant derivatives were randomly picked from plates and incubated in LBHIS medium at 32°C for 16 h with shaking, respectively. Overnight cultures of *C. glutamicum* cells were then transferred into a 100-ml Erlenmeyer flask containing 20 ml of CGXII medium with and without 160 g/L l-lysine at an initial OD_600_ of 0.1. After further incubation for 20 h at 32°C with shaking at 200 rpm, the cell growth was estimated by determining OD_600_ values using a UV–vis spectrophotometer.

## Results

### Comparative transcriptome profiling analyses of *C. glutamicum* responding to high-lysine stress

**FIGURE 1:**
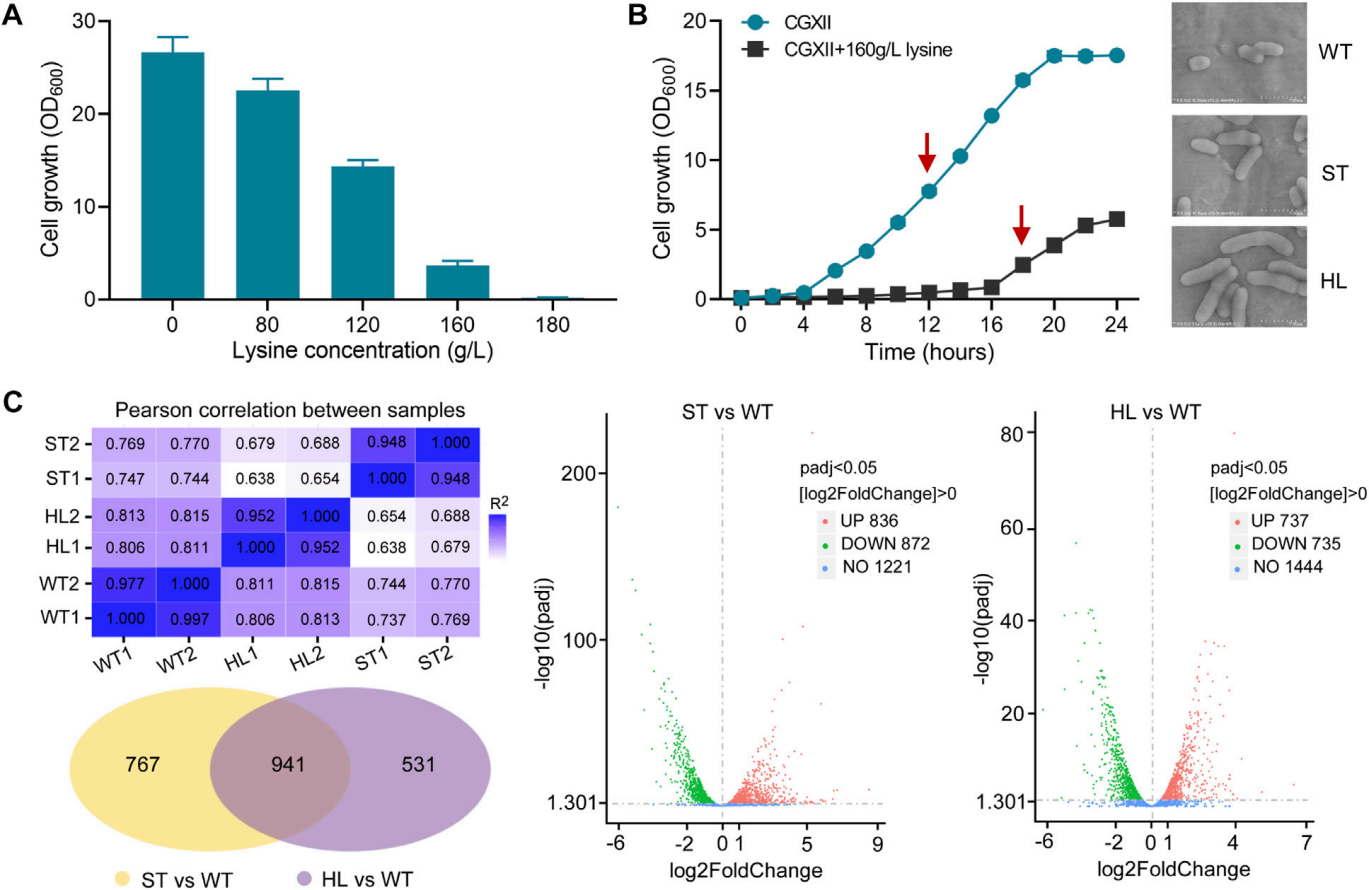
Growth assay and RNA-seq analysis of *C. glutamicum* in response to lysine challenges. **(A)** Growth of *C. glutamicum* cells in CGXII minimum medium with different concentrations of l-lysine. Cells were inoculated into 100 ml flasks containing 20 ml of medium for 20 h at 32°C. **(B)** Growth curves and morphological features of *C. glutamicum* strain with and without l-lysine stress. The growth experiments were carried out in 250 ml flasks containing 100 ml of CGXII medium at 30°C. The arrows indicate time points for RNA harvest at the midexponential growth phase. Scanning electron microscopy (SEM) was used to examine the cell morphology of the WT, ST, and HL samples. **(C)** An overview of transcriptome analysis among different lysine-treated samples. Venn diagram and Volcano plots were used for visualization of differentially expressed genes (DEGs). “WT” represents non-lysine-treated samples, while “HL” indicates cells that suffered from a constant challenge of 160 g/L lysine. “ST” represents the midexponential samples subjected to 160 g/L lysine for 1 h.

During the fermentation process, the accumulation of L-lysine was regarded as a major cause of osmotic stress (Varela et al., 2003; Huang et al., 2021). To explore the possible effects of l-lysine on microbial growth, we measured the growth abilities of the *C. glutamicum* strain at different levels of l-lysine stress. As shown in Figure 1A, high-lysine concentrations obviously reduced the cell growth of *C. glutamicum*, and the strain exhibited only 13.7% of no-stress biomass under stressful condition of 160 g/L l-lysine. Moreover, we obtained growth curves of the *C. glutamicum* strain in CGXII minimum medium with and without high-lysine challenges. In accordance with the aforementioned findings, the growth curves also revealed that high-lysine treatment resulted in extended lag phases and decreased growth rates in *C. glutamicum* (Figure 1B). Additionally, scanning electron microscopy (SEM) observations revealed that the high-lysine adapted cells displayed large elongated morphological features, whereas the control strain had a normal short-rod shape under nonstress conditions (Figure 1B). To gain a better understanding of stress adaptation mechanisms under high-lysine conditions, we performed a comparative transcriptome analysis with samples at the midexponential growth phase. The RNA-seq analysis was designed to encompass multiple samples, including the non-lysine-treated WT control samples, the short-term lysine-treated ST samples, and the long-term lysine-treated HL samples. The high Pearson’s correlation coefficients between two biological replicates indicated that these samples had quite similar overall expression patterns. As shown in Figure 1C, a Venn diagram and Volcano plots were employed to identify the DEGs among three datasets, which revealed that, compared to the non-lysine-treated WT control, 836 genes were upregulated and 872 genes were downregulated in the short-term lysine-treated ST samples, whereas 737 genes were upregulated and 735 genes were downregulated in the long-term lysine-treated HL samples. In addition, a total of 941 overlap DEGs including 269 upregulated genes and 374 downregulated genes relative to the WT control sample were obtained regardless of the exposure time to high-lysine stress. The full list of overlap DEGs is shown in Supplementary Table S2.

### KEGG pathway enrichment analysis of high-lysine-induced stress response

**FIGURE 2:**
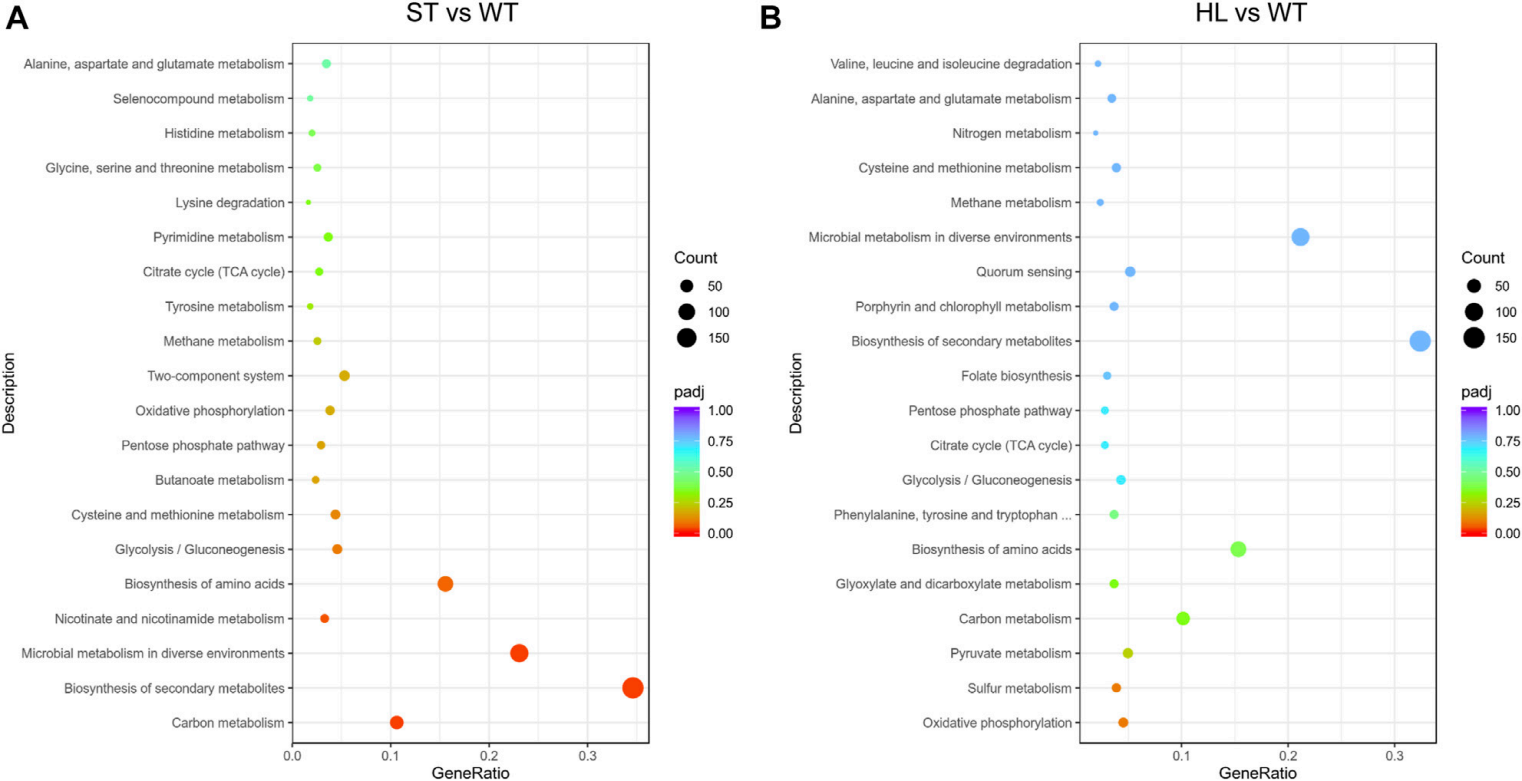
Scatter plot of enriched KEGG pathway analysis in ST **(A)** and HL **(B)** samples compared with the WT control. The *x*-axis represents the rich factor corresponding to each pathway, and the *y*-axis indicates the KEGG pathways. The dot denotes the number of DEGs, and the color reflects the range of a corrected *p*-value (padj). The top 20 most enriched KEGG pathways are shown.

In order to further investigate global transcriptional profiling of high-lysine-induced osmotic stress response, we mapped these identified DEGs to the Kyoto Encyclopedia of Genes and Genomes (KEGG) database. As shown in Figure 2, the top 20 most enriched KEGG pathways in response to high-lysine challenges are listed. The KEGG pathway enrichment results revealed that, when the strain encountered short-term high-lysine stress, the DEGs, especially downregulated genes, were significantly enriched in carbon metabolism (58 genes), biosynthesis of secondary metabolites (189 genes), microbial metabolism in diverse environments (126 genes), and nicotinate and nicotinamide metabolism (18 genes). However, in the long-term high-lysine-treated HL samples, the KEGG pathways exhibiting the most significant changes were enriched in oxidative phosphorylation (21 genes), sulfur metabolism (18 genes), pyruvate metabolism (23 genes), and carbon metabolism (47 genes). Overall, the visualization of the KEGG pathway enrichment of the DEGs indicated that *C. glutamicum* had variable gene expression patterns during different stages of high-lysine stress challenges.

### Global characteristics of metabolic alterations in response to high-lysine stress

**FIGURE 3:**
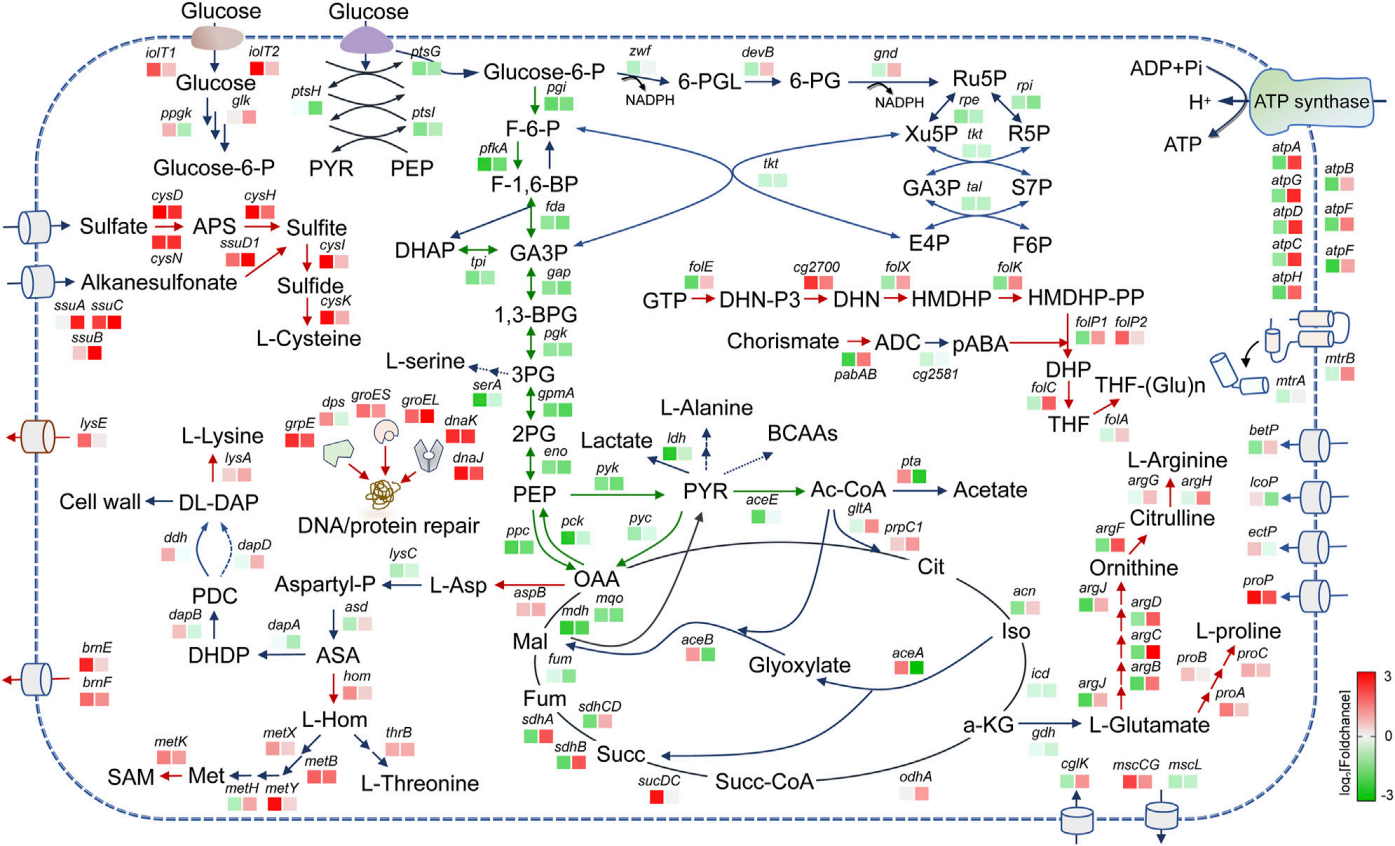
An overview of the major metabolic alterations affected by lysine treatment in *C. glutamicum*. Upregulated and downregulated genes are shown in red and blue, respectively. The color of the boxes represents the log_2_ (fold-change) for genes shown. The left square box represents the short-term lysine-treated ST samples, and the right square box represents the long-term lysine-treated HL samples. Note: glucose-6-P, glucose-6-phosphate; F-6-P, fructose 6-phosphate; F-1,6-BP, fructose 1,6-bisphosphate; DHAP, dihydroxyacetone phosphate; GA3P, glyceraldehyde 3-phosphate; 1,3-BPG, 1,3-bisphosphoglycerate; 3 PG, 3-phosphoglycerate; 2 PG, 2-phosphoglycerate; PEP, phosphoenolpyruvic acid; PYR, pyruvate; Ac-CoA, acetyl coenzyme A; 6-PGL, 6-phosphogluconolactone; 6-PG, 6-phosphogluconate; Ru5P, d-ribulose 5-phosphate; Xu5P, d-xylulose 5-phosphate; R5P, ribose 5-phosphate; S7P, sedoheptulose 7-phosphate; E4P, d-erythrose 4-phosphate; Cit, citrate; Iso, isocitrate; a-KG, alpha-ketoglutaric acid; Succ-CoA, succinyl coenzyme A; Succ, succinate; Fum, fumarate; Mal, l-malate; OAA, oxaloacetic acid; l-Asp, l-aspartate; Aspartyl-P, L-4-aspartyl phosphate; ASA, l-aspartate 4-semialdehyde; L-Hom, l-homoserine; DHDP, 2,3-dihydrodipicolinate; PDC, delta1-piperidine-2,6-dicarboxylate; DL-DAP, meso-diaminoheptanedioate; Met, l-methionine; SAM, S-adenosyl-l-methionine; APS, adenosine 5′-phosphosulfate; GTP, guanosine 5′-triphosphate; DHN-P3, 7,8-Dihydroneopterin 3′-triphosphate; DHN, 7,8-dihydroneopterin; HMDHP, 6-(hydroxymethyl)-7,8-dihydropterin; HMDHP-PP, 6-hydroxymethyl-7,8-dihydropterin diphosphate; DHP, dihydropteroate; THF, tetrahydrofolate; ADC, 4-amino-4-deoxychorismate; pABA, p-aminobenzoate; *pgi*, glucose-6-phosphate isomerase; *pfkA*, 6-phosphofructokinase; *fda*, fructose-bisphosphate aldolase; *tpi*, triosephosphate isomerase; *gap*, glyceraldehyde 3-phosphate dehydrogenase; *pgk*, phosphoglycerate kinase; *gpmA*, phosphoglyceromutase; *eno*, enolase; *pck*, phosphoenolpyruvate carboxykinase; *ppc*, phosphoenolpyruvate carboxylase; *pyk*, pyruvate kinase; *serA*, phosphoglycerate dehydrogenase; *ioIT1/2*, inositol transport protein; *ptsG*, beta-glucoside PTS system EIICBA component; *ptsH*, phosphocarrier protein HPr; *ptsI*, phosphoenolpyruvate-protein phosphotransferase; *ppgk*, polyphosphate glucokinase; *glk*, glucokinase; *zwf*, glucose-6-phosphate dehydrogenase; *devB*, 6-phosphogluconolactonase; *gnd*, 6-phosphogluconate dehydrogenase; *rpe*, ribulose-phosphate 3-epimerase; *rpi*, ribose 5-phosphate isomerase; *tkt*, transketolase; *tal*, transaldolase; *ldh*, l-lactate dehydrogenase; *aceE*, pyruvate dehydrogenase E1 component; *gltA*, citrate synthase; *pta*, phosphate acetyltransferase; *prpC1*, 2-methylcitrate synthase; *acn*, aconitate hydratase; *icd*, isocitrate dehydrogenase; *odhA*, 2-oxoglutarate dehydrogenase; *sucDC*, succinyl-coA synthetase; *sdhCDAB*, succinate dehydrogenase CDAB; *fum*, fumarate hydratase; *mdh*, malate dehydrogenase; *mqo*, malate dehydrogenase; *aceA*, isocitrate lyase; *aceB*, malate synthase; *aspB*, aspartate aminotransferase; *lysC*, aspartokinase; *asd*, aspartate-semialdehyde dehydrogenase; *hom*, homoserine dehydrogenase; *thrB*, homoserine kinase; *metX*, homoserine acetyltransferase; *metB*, cystathionine gamma-synthase; *metY*, O-acetylhomoserine (thiol)-lyase; *metH*, methionine synthase; *metK*, S-adenosylmethionine synthase; *dapA*, dihydrodipicolinate synthase; *dapB*, dihydrodipicolinate reductase; *ddh*, meso-diaminopimelate d-dehydrogenase; *dapD*, tetrahydrodipicolinate succinylase; *lysA*, diaminopimelate decarboxylase; lysE, lysine efflux permease; *brnEF*, branched-chain amino acid exporter; *gdh*, glutamate dehydrogenase; *argJ*, glutamate N-acetyltransferase; *argB*, acetylglutamate kinase; *argC*, N-acetyl-gamma-glutamyl-phosphate reductase; *argD*, acetylornithine aminotransferase; *argF,* ornithine carbamoyltransferase; *argG*, argininosuccinate synthase; *argH*, argininosuccinate lyase; *cglK*, K^+^ transport protein; *mscS*, mechanosensitive channel protein; *mscL*, mechanosensitive channel protein; *proA*, glutamate-5-semialdehyde dehydrogenase; *proB*, glutamate 5-kinase; *proC*, pyrroline-5-carboxylate reductase; *betP*, glycine betaine transporter; *lcoP*, ectoine betaine transporter; *ectP*, ectoine/proline/glycine betaine carrier EctP; *proP*, proline/betaine transporter; *atpABCDFGH*, ATP synthase subunits; *folE*, GTP cyclohydrolase 1; *cg2700*, alkaline phosphatase; *folX*, 7,8-dihydroneopterin aldolase; *folK*, 2-amino-4-hydroxy-6-hydroxymethyldihydropteridine diphosphokinase; *folP1*, dihydropteroate synthase; *folp2*, dihydropteroate synthase; *cg2581*, glucosyl-3-phosphoglycerate phosphatase; *pabAB*, para-aminobenzoate synthetase; *folC*, dihydrofolate synthase; *folA*, dihydrofolate reductase; *dps*, starvation-induced DNA protecting protein; *grpE/groES/groEL/dnaK/dnaJ,* molecular chaperone; *cysD,* sulfate adenylyltransferase; *cysH,* phosphoadenosine phosphosulfate reductase; *cysI,* sulfite reductase; *cysK,* cysteine synthase; *ssuD1,* alkanesulfonate monooxygenase; *cysN,* sulfate adenylyltransferase; and *ssuCBA,* ABC-type aliphatic sulfonate transporter.

To obtain a broader overview of the expression profiles of genes involved in cellular metabolic processes, we analyzed global changes in gene expression under short-term and long-term high-lysine stress conditions. As shown in Figure 3, the high levels of l-lysine resulted in global metabolic alterations of *C. glutamicum*, and some types of stress responses may enable it to survive the prolonged periods of high-lysine osmotic stress. In accordance with growth-inhibitory phenotypes, the presence of high-lysine stress obviously decreased the expression of many key genes involved in the central carbon metabolism pathways, including glycolysis, pentose phosphate pathway (PPP), and the tricarboxylic acid (TCA) cycle. However, the non-phosphotransferase (PTS) glucose uptake system consisting of *iolT1*, *iolT2*, *glk*, and *ppgk* was unexpectedly induced, which might be attributed to a synergistic compensatory response for efficient utilization of sugar substrates during stress. Additionally, in the HL samples that were subjected to long-term high-lysine stress treatment, some genes such as *devB*, *gnd*, *gltA*, *acn*, *odhA*, and *sdhCDAB* that were important for the PPP pathway and TCA cycle showed varying but enhanced levels of expression, which were believed to contribute to generating the building blocks and energy and reducing equivalents for maintaining cell metabolism and growth.

Apart from carbon metabolism, the biosynthesis of specific amino acids was also affected in the short-term ST and long-term HL samples. For example, the expressions of enriched genes implicated in l-proline and l-arginine biosynthetic pathways were clearly enhanced in the HL samples. In addition, we also found in agreement with some previous reports that the addition of exogenous proline or arginine could contribute to improving high-lysine stress tolerance (Supplementary Figure S1). Given that these two amino acids were reported to be important compatible solutes in response to osmotic stress (Wood, 2011; Guan et al., 2017), we speculated that upregulation of the related genes may also help protect cells from long-term high-lysine-induced osmotic stress. As for the decreased biosynthesis of l-arginine in the ST samples, it is possibly due to the adverse metabolic disorders that occur when cells have encountered a stressful environment. In addition, many genes involved in the biosynthesis of the aspartic acid family of amino acids such as l-threonine and l-methionine were also induced under stress conditions. One possible explanation for this was that the expression of these genes may be a compensatory adaptation to alleviate the feedback inhibition of lysine accumulation on key enzyme activities in the aspartic acid metabolic pathway. As expected, the expression levels of l-lysine transporter genes *lysE* and *brnEF* were significantly increased, thus facilitating the efflux of toxic lysine out of the cell under high-lysine stress conditions.

Folate and its derivatives serve as coenzyme forms in supporting acceptance, redox processing, and transfer of one-carbon units in cellular metabolic pathways (Bailey and Gregory, 1999; Danchin et al., 2020). The analyses showed that some genes of the folate biosynthetic pathway were induced by the long-term lysine treatment, implying the possibility that the biosynthesis of folate was beneficial for survival under high-lysine conditions. Sulfur metabolism is reported to be essential for the production of various sulfur-containing biomolecules and plays critical roles in many redox reactions for maintaining biological functions (Mukwevho et al., 2014; Miller and Schmidt, 2020). The analyses also revealed that sulfur assimilation pathways were always activated in the stress-treated samples regardless of the exposure time to high-lysine challenges. In addition, we also observed increased expressions of molecular chaperone-related genes including *grpE*, *groES*, *danK*, and *dnaJ*, supporting the current opinion that such DNA/protein repair systems may protect cells from additional stress-induced damages.

In previous studies, researchers have reported that several transporters such as BetP, LcoP, EctP, ProP, and CglK constitute compatible solute uptake systems and are associated with the responses and adaptation to osmotic stress in *C. glutamicum* (Krämer and Morbach, 2004; Krämer, 2009; Ochrombel et al., 2011; Bremer and Krämer, 2019). However, except for the *proP* gene, the expression of other genes demonstrates no obvious differences under stress conditions. In addition, the trehalose biosynthetic pathways were also not affected in stress-treated samples compared to the WT control groups. These findings imply that these aforementioned compatible solutes may play a minor role in the high-lysine stress response.

### Growth stress assays to investigate gene targets conferring high-lysine tolerance

**FIGURE 4:**
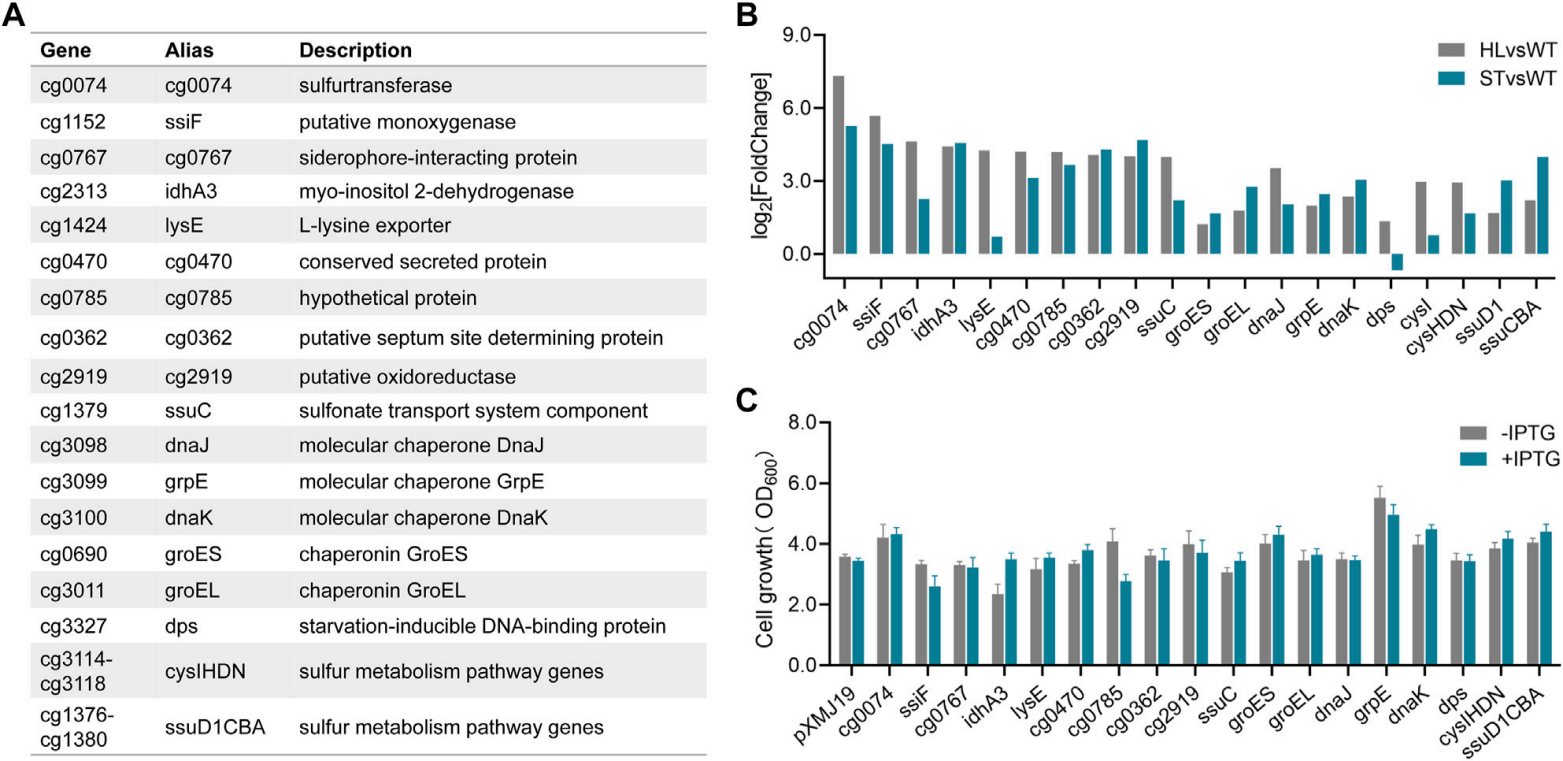
Effects of some representative DEGs on conferring high-lysine tolerance. **(A)** A summary of 18 differentially expressed high-lysine-related genes. **(B)** Relative expression levels of 18 DEGs in selected samples based on RNA-seq data. **(C)** Cell growth of *C. glutamicum* recombinant strains containing the control plasmid pXMJ19 or its derivatives under stressful conditions with or without IPTG addition. Then, 160 g/L lysine was used to mimic osmotic stress that occurs during the fermentation process.

According to the global gene expression analysis in Figure 3, the enriched genes involved in some cellular metabolic processes were obviously upregulated under both short-term and long-term high-lysine stress conditions, especially DNA/protein repair systems and sulfur metabolism. To explore the potential roles of such gene targets in protection against high-lysine stress, we selected six upregulated representative DEGs (*dnaJ*, *grpE*, *dnaK*, *groES*, *groEL*, and *dps*) encoding molecular chaperones and two gene clusters (*cysIHDN* and *ssuD1CBA*) involved in sulfur transport and utilization for high-lysine tolerance assays (Figure 4A). In addition, the 10 top upregulated DEGs from the long-term RNA-seq data (*cg0074*, *ssiF*, *cg0767*, *idhA3*, *lysE*, *cg0470*, *cg0785*, *cg0362*, *cg2919*, and *ssuC*) were also included for the selection of promising gene targets to improve high-lysine-induced stress tolerance (Figure 4A). The expression levels of the abovementioned genes in the short-term ST and long-term HL samples compared with the nonstress control were obtained from RNA-seq analyses and are shown in Figure 4B. To address these concerns, we therefore constructed a series of expression plasmids to investigate their effects on high-lysine stress tolerance. As shown in Figure 4C, the increased expressions of *grpE*, *cg0074*, *dnaK, groES, cysIHDN*, and *ssuD1CBA* exhibited varying degrees of growth improvement under high-lysine osmotic stress conditions. In particular, the introduction of molecular chaperon grpE showed up to a 40% increase in biomass under the tested stress conditions, thus supporting its potential role in conferring high-lysine tolerance.

### Investigation of DNA damage repair in lysine-induced osmotic stress

**FIGURE 5:**
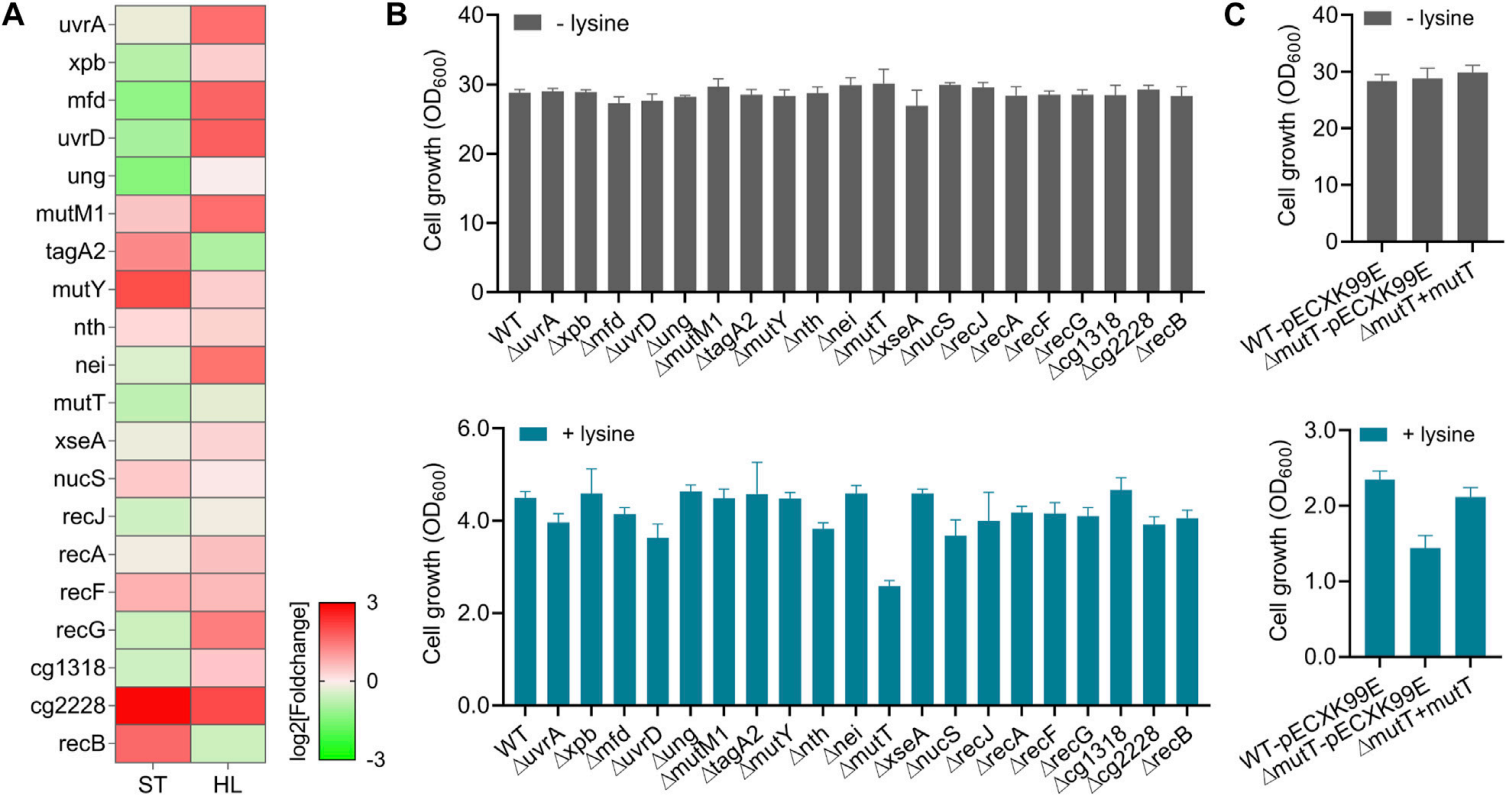
Roles of DNA repair systems in protecting cells against high-lysine-induced osmotic stress. **(A)** Expression profiles of representative genes implicated in DNA repair mechanisms among ST and HL samples. **(B)** Growth assays of the wild-type and DNA repair-defective mutants with and without lysine treatment. After overnight pregrowth at 32°C, the cells were, respectively, shifted to CGXII medium with an initial OD_600_ of 0.1 and incubated for another 20 h with shaking. Then, 160 g/L lysine was used to mimic osmotic stress. **(C)** Growth assays of the wild-type, *∆mutT,* and complemented strains in response to the challenge of 160 g/L lysine. The shuttle vector pECXK-99E was used for the expression of *mutT* gene in *C. glutamicum*.

The maintenance of genome integrity is critical for cell survival and proper cellular function. It has been shown that the microbes can employ DNA damage responses to maintain genome integrity and prevent harmful mutations, which facilitate and confer resistance and adaptation to stress-inducing factors (Kreuzer, 2013; Guan and Liu, 2020). In one example, previous reports showed that the DNA repair protein RecO was a promising target in enhancing the tolerances of lactic acid bacteria against multiple stresses (Wu et al., 2013; Zhang et al., 2014). Given the importance of DNA repair pathways in stress adaptations, we also analyzed the expressions of genes involved in DNA damage response and investigated their potential functions in response to high-lysine stress. These representative DEGs covered the main DNA repair pathways in *C. glutamicum* (Resende et al., 2011; Kautzmann and Altenbuchner, 2017), including nucleotide excision repair NER (*uvrA*, *xpb*, *mfd*, and *uvrD*), base excision repair BER (*ung*, *mutM1*, *tagA2*, *mutY*, *n*th, and *nei*), mismatch repair MMR (*mutT*, *xseA*, *nucS*, and *recJ*), recombination repair RER (*recA*, *recF*, and *recG*), and other repair systems (*cg1318*, *cg2228*, and *recB*). The RNA-seq expression levels of the abovementioned genes in tested short-term lysine-treated ST and long-term lysine-treated HL samples are represented in Figure 5A, revealing that most repair genes appeared to be activated in response to high-lysine-induced osmotic stress.

In order to investigate the potential functions of these gene targets in high-lysine-induced stress response, we performed stress tolerance assays between the wild-type and corresponding mutant strains. As shown in Figure 5B, the absence of certain DNA repair-related genes, such as *mutT* encoding an 8-oxo-dGTP diphosphatase involved in DNA mismatch repair, clearly reduced cell growth under high-lysine stress conditions (Figure 5B). To further confirm the possible role of *mutT* gene in stress response, we constructed a plasmid-based *mutT-*complemented strain. As shown in Figure 5C, the *∆mutT* mutant exhibited comparable growth to that of wild-type and complemented strains under no-stress conditions. However, when encountering high-lysine challenges, the growth ability of the *∆mutT* mutant was retarded, and this growth defect could largely be rescued by the reintroduction of the *mutT* gene.

Guanosine nucleotides are particularly susceptible to oxidative damage during stress response, leading to the formation of oxidized guanine products such as 8-oxo-GDP, 8-oxo-GTP, 8-oxo-dGDP, and 8-oxo-dGTP (Kapoor et al., 2019). MutT possesses the ability to hydrolyze 8-oxo-dGTP to monophosphate, reducing the incorporation of 8-oxo-dG into DNA (Taddei et al., 1997). In *E. coli*, *mutT* deletion was reported to increase the spontaneous mutation frequency more than 100-fold above background by enhancing expected A to C or T to G transversion mutations (Dupuy et al., 2020). Consistent with these opinions, we found that *mutT* is required for protecting cells against high-lysine stress in *C. glutamicum*, which may also be largely attributed to the prevention of mutagenic effects of 8-oxo-dG lesions in DNA. However, a seemingly puzzling phenomenon was observed from the RNA-seq data, in which the expressions of *mutT* gene were decreased by 39% and 21% under high-lysine stress conditions. One possibility for such discrepancy is that despite the cells requiring an efficient DNA repair system to rescue mutagenesis phenotypes and stress sensitivity, the proper inhibition of DNA repair activity to increase the frequencies of beneficial mutations may also be a compromise for strains to improve stress resistance. Taken together, these findings showed that the DNA repair component MutT had an important role in response to high-lysine stress, but its expression pattern response to stressful situations still needs to be studied in more detail.

### Investigation of NADH dehydrogenase in lysine-induced osmotic stress

**FIGURE 6:**
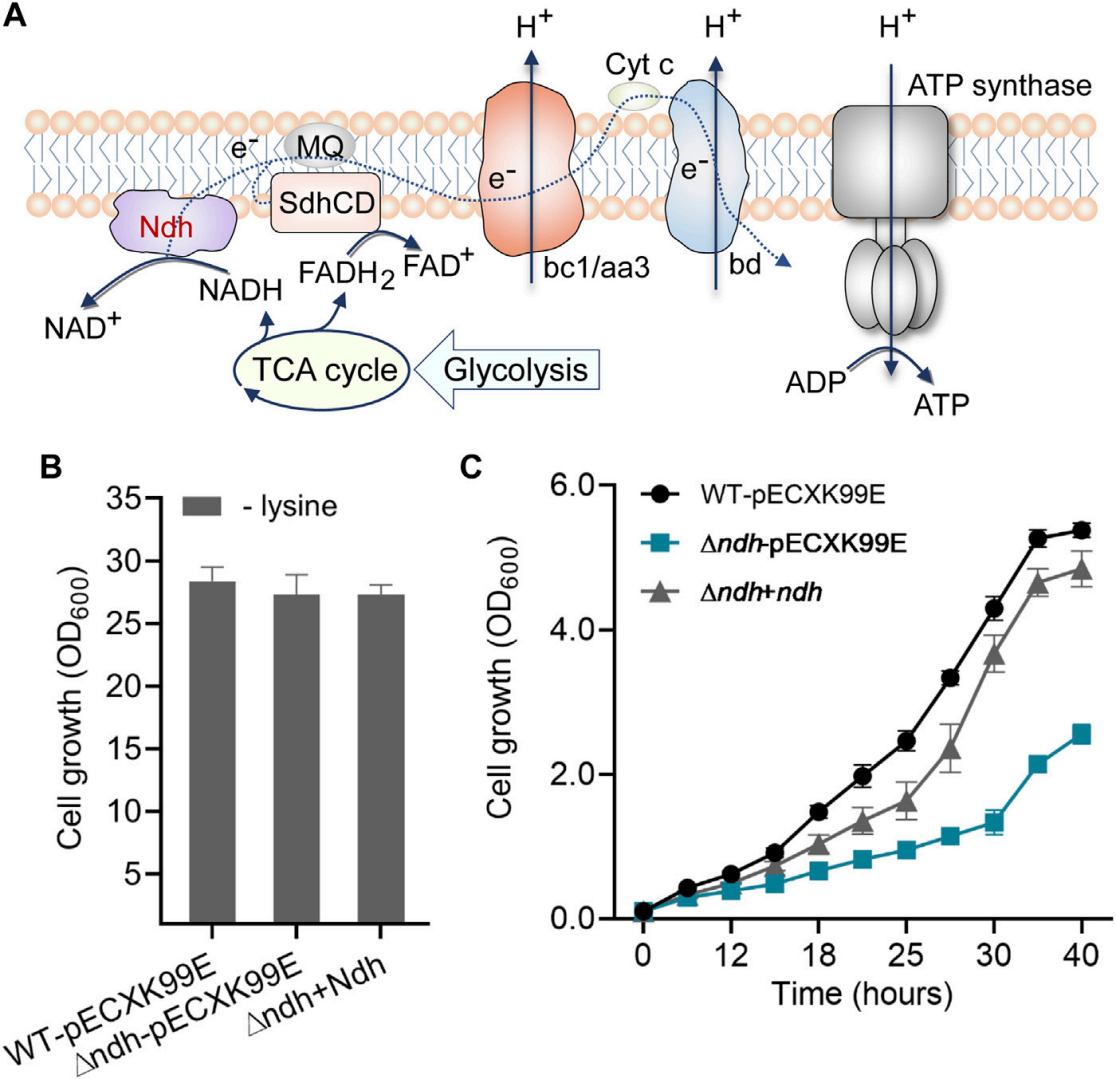
Role of the NADH dehydrogenase Ndh in protecting cells against high-lysine-induced osmotic stress. **(A)** Schematic diagram of Ndh in the oxidative phosphorylation pathway. **(B,C)** Growth assays of the wild-type, *∆ndh*, and complemented strains without or with a high-lysine challenge.

In the comparative analysis of DEGs, we also found that the gene expressions of F_0_F_1_-ATP synthase subunits *atpADGHCBFE* were obviously enhanced in response to long-term high-lysine stress (Figure 3C), implying the importance of energy generation in stress tolerance. Generally, the supply and consumption of energetic cofactors in metabolism are central concerns for increasing carbon and energy flexibility (Liu et al., 2018). NADH dehydrogenase represents a main entry site for reducing equivalents from central metabolism into the respiratory chain (Nantapong et al., 2004; Nantapong et al., 2005; Zelle et al., 2021). *C. glutamicum* has been reported to possess one non-proton-pumping, single-subunit NADH dehydrogenase Ndh (*cg1656*) and lack the proton-pumping complex I (Figure 6A) (Bott and Niebisch, 2003). RNA-seq analysis from the ST and HL samples revealed that the high-lysine stresses did not affect the expression levels of *ndh* genes in *C. glutamicum*. Further physiological experiments showed that deletion of *ndh* gene did not cause serious effects on cell growth under the tested no-stress condition, which was consistent with previous reports (Nantapong et al., 2004; Maeda et al., 2021) (Figure 6B). However, under high-lysine stress conditions, the growth curve analysis suggested that the *∆ndh* mutant showed clear growth defects as compared with wild-type and complemented strains. Relative to wild-type controls, the complemented strains eventually exhibited a near-similar growth phenotype despite lagging growth in the early stages. Overall, these findings revealed that the NADH oxidation system component Ndh was required for enhancing high-lysine stress tolerance in *C. glutamicum*.

## Discussion

Nowadays, *C. glutamicum* is extensively used as the preferred cell factory for the industrial production of various amino acids such as l-lysine and l-glutamate (Becker et al., 2018; Wendisch, 2020). With the development of efficient microbial cell factories through system metabolic engineering, the high-level accumulation of final products generally tends to trigger intracellular osmotic stress (Mukhopadhyay, 2015), which in turn adversely affects cell growth and production levels. In recent years, more and more efforts have focused on tolerance engineering for developing robust strains with improved properties toward toxic or inhibitory end products. To establish this property, we first require the identification of stress-responsive functional and regulatory elements. Currently, the promising targets that can be used for improving the tolerance and robustness of industrial microorganisms include molecular chaperones, transporters, transcription regulators, cell membranes, macromolecule repair systems, and reactive oxygen species (ROS)-scavenging enzymes(Mukhopadhyay, 2015; Qi et al., 2019; Liu et al., 2021), which provide the foundation for strain improvement through synthetic biology.

Previous studies have revealed that some transport systems are highly related to osmoregulation in bacteria (Wood, 2007; Krämer, 2009). For example, *C. glutamicum* is reported to have five osmoregulated secondary carriers for the uptake of compatible solutes, namely, BetP, EctP, LcoP, PutP, and ProP. The potassium channel CglK is required for the accumulation of potassium levels, which contributes to the bacterial response to osmotic stress challenges (Ochrombel et al., 2011). Unfortunately, the results from our transcriptome studies showed that the expression differences of the abovementioned genes, except *proP*, were not statistically significant under high-lysine-induced osmotic stress conditions. Interestingly, the biosynthetic pathways of l-arginine and l-proline, also referred to as compatible solutes or osmoprotectants, were activated in response to long-term high-lysine challenges, supporting that the accumulation of intracellular l-arginine and l-proline may offer favorable benefits for high-lysine stress adaptation. In addition, the expressions of many DEGs enriched in sulfur metabolism pathways were also found to be induced under stress conditions, supporting the importance of regulating sulfur-mediated redox homeostasis in response to high-lysine stress.

The MtrB–MtrA two-component system of *C. glutamicum* is thought to be involved in osmosensing and osmoregulation (Möker et al., 2007; Bott and Brocker, 2012). MtrB served as a sensor for environmental stimuli, and the response regulator MtrA is found to activate the expression of several target genes, including *proP*, *betP*, and *lcoP*. In addition, the mechanosensitive channel, including MscL and an MscS-like MscCG of *C. glutamicum* ATCC 13032, has been characterized as an osmoregulator for adaptation to osmotic stress (Nakayama et al., 2013; Nakayama, 2021). Apart from this, MscCG was also identified as a major l-glutamate exporter, which can also serve as osmolytes, helping cells survive osmotic stress (Nakayama et al., 2018; Kawasaki and Martinac, 2020). Through the analysis of the transcriptional profiling of these aforementioned genes, as shown in Figure 3, we found that despite the expression levels of *mtrB* and *mtrA* genes showing approximately 30% reduction in the short-term lysine-treated ST samples, the *mtrB* level was increased up to two-fold when encountered with long-term high-lysine stress. Moreover, differential regulation patterns occurred at *mscCG* and *mscL* genes under high-lysine conditions, and *mscCG* displayed a consistently higher expression level. These findings further highlight the potential functions of the MtrB sensor and MscCG exporter in high-lysine osmoprotection.

In this study, we also conducted stress tolerance assays to identify DEGs that are essential for protecting against high-lysine-induced osmotic stress. The results showed that the introduction of several chaperone genes especially *grpE* contributed to the improvement of high-lysine--induced osmotic stress adaptation. Additionally, we found that the DNA repair component MutT and energy-transducing NADH dehydrogenase Ndh also played critical roles in protecting cells against osmotic stress caused by high lysine concentrations. The absence of these genes dramatically attenuated the growth abilities of *C. glutamicum* cells under stress conditions. In conclusion, our studies have aimed at providing a deeper understanding of the global physiological changes of *C. glutamicum* in response to high-lysine challenges. These findings elucidate the importance of molecular chaperones, DNA repair enzymes, and energy supply-related proteins, with the goal of providing new promising targets for the engineering of microbial stress tolerance against harsh environmental conditions during l-lysine fermentation.

## Data Availability

The datasets presented in this study can be found in online repositories. The names of the repository/repositories and accession number(s) can be found below: SRA PRJNA835314.
