# Supplementary material for: Transcriptome profiles of high-lysine adaptation reveal insights into osmotic stress response in Corynebacterium glutamicum
Source: Front Bioeng Biotechnol. 2022 Aug 9;10:933325. doi: 10.3389/fbioe.2022.933325 (PMC9395588; doi:10.3389/fbioe.2022.933325)
Supplement: Supplementary file 1 [file DataSheet1.DOCX]

**Frontiers in Bioengineering and Biotechnology**

**Supplementary Material**

**Transcriptome profiles of high-lysine adaptation reveal insights into osmotic stress response in *Corynebacterium glutamicum***

Jian Wang^1^, Jian Yang^1^, Guoxin Shi^1^, Weidong Li^1^, Yun Ju^2^, Liang Wei^3^, Jun Liu^3,4^, Ning Xu^3,4*^

^1^ College of Biological and Agricultural Engineering, Jilin University, Changchun 130022, China,

^2^ School of Food Engineering and Biotechnology, Tianjin University of Science and Technology, Tianjin 300457, P. R. China.

^3^ Tianjin Institute of Industrial Biotechnology, Chinese Academy of Sciences, Tianjin 300308, China,

^4^ Key Laboratory of Systems Microbial Biotechnology, Chinese Academy of Sciences, Tianjin 300308, China

Running title: Transcriptome profiles for high-lysine stress response in *C. glutamicum*

* Corresponding authors:

[xu_n@tib.cas.cn](mailto:xu_n@tib.cas.cn)


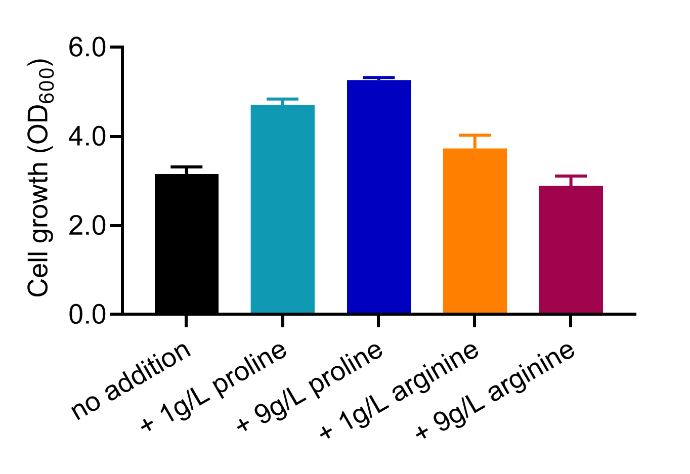


Figure S1. Effects of proline or arginine addition on protecting cells against high-lysine stress. 160 g/L lysine was used to mimic osmotic stress that occurs during the fermentation process.
